# Genome-Wide Identification of the Bcl-2 Associated Athanogene (BAG) Gene Family in *Solanum lycopersicum* and the Functional Role of *SlBAG9* in Response to Osmotic Stress

**DOI:** 10.3390/antiox11030598

**Published:** 2022-03-21

**Authors:** Hailong Jiang, Yurong Ji, Jiarong Sheng, Yan Wang, Xiaoya Liu, Peixiang Xiao, Haidong Ding

**Affiliations:** Joint International Research Laboratory of Agriculture and Agri-Product Safety of Ministry of Education of China, College of Bioscience and Biotechnology, Yangzhou University, Yangzhou 225009, China; jhl19923574146@163.com (H.J.); jyr0806@163.com (Y.J.); myyjjfb@163.com (J.S.); wy1123406945@163.com (Y.W.); arielnxy@outlook.com (X.L.); xiaopeixiang_1998@163.com (P.X.)

**Keywords:** antioxidant defense, arabidopsis, bioinformatic analysis, *SlBAG* genes, *SlBAG9*, *Solanum lycopersicum*

## Abstract

The Bcl-2-associated athanogene (BAG) proteins are a family of multi-functional group of co-chaperones regulators, modulating diverse processes from plant growth and development to stress response. Here, 10 members of *SlBAG* gene family were identified based on the available tomato (*Solanum lycopersicum*) genomic information and named as *SlBAG1-10* according to their chromosomal location. All SlBAG proteins harbor a characteristic BAG domain, categorized into two groups, and SlBAG4, SlBAG7, and SlBAG9 of group I contain a plant-specific isoleucine glutamine (IQ) calmodulin-binding motif located in the N terminus. The quantitative real-time PCR expression analysis revealed that these *SlBAG* genes had organ-specific expression patterns and most *SlBAG* genes were differentially expressed in multiple abiotic stresses including drought, salt, high temperature, cold, and cadmium stress as well as abscisic acid and H_2_O_2_. In addition, heterologous overexpression of *SlBAG9* increased the sensitivity of Arabidopsis to drought, salt, and ABA during seed germination and seedling growth. The decreased tolerance may be due to the downregulation of stress-related genes expression and severe oxidative stress. The expression levels of some stress and ABA-related genes, such as *ABI3*, *RD29A*, *DREB2A*, and *P5CS1*, were significantly inhibited by *SlBAG9* overexpression under osmotic stress. Meanwhile, the overexpression of *SlBAG9* inhibited the expression of *FSD1* and *CAT1* under stress conditions and the decreased levels of superoxide dismutase and catalase enzyme activities were detected accompanying the trends in the expression of both genes, which resulted in H_2_O_2_ accumulation and lipid peroxidation. Taken together, these findings lay a foundation for the future study of the biological function of *SlBAG* genes in tomato.

## 1. Introduction

B cell lymphoma 2 (Bcl-2)-associated athanogene (BAG) protein is a relatively conservative protein in animals and plants. BAG proteins share a common conserved BAG domain (BD), which interacts with the ATPase domain of heat-shock protein 70 (Hsc70/Hsp70 in the C-terminal region but is generally different in the N-terminal region, which gives specificity to specific proteins and pathways [1]. In animals, BAGs are widely involved in many biological processes, such as tumor regulation, apoptosis and stress response [1,2]. In plants, seven BAG family genes were first discovered in Arabidopsis [3,4]. The family of plant BAG protein can be divided into two categories. The first category contains N-terminal ubiquitin-like (UBL) domain and BAG domain, including AtBAG1–4 proteins. They may be direct homologues of animal BAG1, with high similarity in structure and function. The second type (BAG5-7 proteins) contains an isoleucine glutamine (IQ) motif binding to Ca^2+^-free calmodulin (CaM) near BAG domain, which is unique to plants [3]. Plant BAG proteins are involved in biological processes such as plant programmed cell death (PCD) and autophagy, and play an important role in plant response to abiotic stresses such as salt, drought, temperature, and pathogen infection. Some BAG family genes have been explored in other plants such as rice, tomato, and wheat [5,6,7,8,9], but presently, as a model plant Arabidospis has gained the primary research attention, while relatively little is known about the functional research on other species.

The Arabidopsis seedlings overexpressing *AtBAG1* challenged with salinity stress and decreased seedling growth [10]. The *atbag4* mutant plants were more sensitive to salt stress, while the tobacco plants overexpressing *AtBAG4* gene showed stronger tolerance to ultraviolet (UV), low temperature stress, oxidative stress, drought, and salt stress than the wild type [3]. AtBAG1-AtBAG4 bound to Hsc70 through the BAG domain. The *atbag5* mutant showed the delayed aging, while the overexpression transgenic lines showed the premature aging. As a signaling hub, AtBAG5 linked the Ca^2+^ signaling network with the Hsc70 chaperone system to regulate plant senescence [11,12]. AtBAG6 cleavage triggered autophagy and plant defense and AtBAG6 [13]. AtBAG7 was an endoplasmic reticulum (ER) localization protein that played a central regulatory role in the heat-induced unfolded protein response (UPR) pathway [14]. The double-faced role of AtBAG7 in plant–phytophthora interaction has been found recently [15]. However, it is gratifying that, nowadays, studies on other plants species are becoming available. Transgenic rice plants overexpressing *OsBAG4* showed tolerance to NaCl stress [16]. OsBAG4 is an active regulator of disease resistance and a rice E3 ubiquitin ligase EBR1 targeted OsBAG4 for proteasome degradation [17]. Soybean *GmBAG6a* gene overexpressed in Arabidopsis had the ability to resist nematode infection [18]. Overexpressing BAG family gene *HSG1* from grapes in Arabidopsis plants showed obvious resistance to high temperature [19]. Overexpression of wheat *TaBAG2* increased Arabidopsis heat tolerance [6]. *SlBAG2* and *SlBAG5b* mediated tomato leaf tolerance to dark stress and senescence [8].

Based on the publication of plant genome sequence databases, the BAG gene family have been proved to exist in different species, such as *Arabidopsis thaliana* [3], *Oryza sativa* [5,20], and *Physcomitrium patens* [21]. We have previously tried to explore the key high-temperature-responsive genes in tomato using integrative analysis of transcriptome and proteome [22] and *SlBAG9* with a high expression level under high temperature stress was screened at the transcriptional and protein levels, which aroused our interest in the BAG gene family. In this study, ten *SlBAG* genes were identified, the related characteristics of *SlBAG* gene and protein were studied, and the gene expression pattern under different stress conditions were explored. In addition, further research on the function of *SlBAG9* was carried out. Heterologous overexpression of *SlBAG9* in Arabidopsis increased the sensitivity of Arabidopsis to drought, salt, and abscisic acid (ABA), which was reflected in the decrease in seed germination rate and seedling growth, low expression levels of stress/ABA-responsive genes and activities of superoxide dismutase (SOD) and catalase (CAT), and aggravated oxidative damage. Taken together, these findings lay a foundation for the future study of the biological function of *SlBAG* genes in tomato.

## 2. Materials and Methods

### 2.1. Genome-Wide Identification and Analysis of the Tomato BAG Gene Family

To identify the tomato BAG gene family, the BAG domain (PF02179) was used as a query to perform an HMMER (v3.3.2) search against the tomato proteome downloaded from Tomato Genome Annotation ITAG4.0 database (https://solgenomics.net, accessed on 10 October 2021). At the same time, a comprehensive search was carried out by using the amino acid sequence of *Arabidopsis thaliana* according to a previous study [4], of which seven AtBAG proteins were obtained from the *Arabidopsis thaliana* Araport11. The reliability of *SlBAG* gene sequences and genome sequences was further confirmed by searching the NCBI database (https://www.ncbi.nlm.nih.gov, accessed on 20 October 2021). The genomic regions, transcripts, and products were from Tomato Genome version SL4.0 (https://solgenomics.net, accessed on 10 October 2021) and NCBI data comparison. The molecular weight (Mw) and isoelectric point (pI) were calculated by ProtParam on the ExPASy Server (https://web.expasy.org/protparam, accessed on 25 October 2021).

### 2.2. Phylogenetic Analysis

The BAG protein sequences were downloaded from database *Solanum lycopersicum* ITAG4.0, *Arabidopsis thaliana* Araport11, and *Oryza sativa* v7.0, respectively. Multiple sequence alignments of BAG proteins were carried out using MUSCLE and the tree was constructed by PhyML method (phylogeny.fr/simple_phylogeny.cgi, accessed on 5 November 2021).

### 2.3. Motif Analysis of SlBAG Proteins

The conserved motifs of tomato SlBAGs were predicted by the MEME tool (Version 5.4.1, http://meme-suite.org/tools/meme, accessed on 11 December 2021). The number of BAG conserved domain was validated using NCBI conserved domains (CDD) and shown using the HMMER web server (Biosequence analysis using profile hidden Markov Models|HMMER).

### 2.4. Gene Structure and Cis-Element Analysis of SlBAG Genes

The Gene Structure Display Server (http://gsds.cbi.pku.edu.cn, accessed on 15 December 2021) was used for the gene structure determination. The cis-regulatory elements of the SlBAG gene promoters were analyzed by the PLANTCARE database (http://bioinformatics.psb.ugent.be/webtools/plantcare/html, accessed on 20 December 2021) and the elements were displayed on the TBtool software (version v1.0986853, https://github.com/CJ-Chen/TBtools, accessed on 28 December 2021).

### 2.5. Expression Analysis of SlBAG Genes

Seeds of *Solanum lycopersicum* cv. Ailsa Craig were sterilized and sown in the solid Murashige and Skoog (MS) medium at 25 °C/20 °C, and 20-day-old seedlings were used for the following treatments. For *SlBAG* gene expression under stresses, tomato seedlings were cultured in liquid MS medium for 24 h, then exposed to salt, drought, high temperature, cold, cadmium, H_2_O_2_, and ABA treatment for 1, 3, 6, 12, 24, and 48 h. Tomato plants were transferred to liquid medium containing 100 mM NaCl, 20% polyethylene glycol-6000, 50 uM cadmium, 100 uM ABA, and 10 mM H_2_O_2_, respectively.

For HT and cold, the seedlings were exposed to HT (42 °C) or 4 °C conditions. All real leaves were collected, frozen, and preserved at −70 °C for RNA extraction. For organs-specific expression of *SlBAG* genes, organs from root, stem, leaf, flower, green fruit, and red fruit of different stages were collected according to the method of Ding et al. [23]. The transcription levels of *SlBAG* genes were determined by quantitative real-time PCR (qRT-PCR).

### 2.6. Phenotypic Analysis of SlBAG9 Overexpression Plants under Osmotic Stress

To analyze the phenotype of *SlBAG9*-overexpressing plants under osmotic stress (drought and salt), two developmental stages were used for processing. For seed germination assay, the seeds of *Arabidopsis thaliana* ecotype Columbia-0 (WT) and *SlBAG9* overexpression line 2–12 and 4–9 [22] were surface-sterilized and sowed on 1/2 MS plates supplemented with or without 200 and 300 mM mannitol (M1, M2), 175 and 200 mM NaCl (N1, N2), and 1.0 and 1.5 µM ABA (A1, A2), and placed under 4 °C for 3 days, then moved to a growth room under a 16 h-light/8 h-dark photoperiod in the greenhouse. The seed germination rate (root emergence) was evaluated every day, and the number of seedlings with significantly expanded and green cotyledons were also evaluated. For seedling growth assay, the seven-day-old seedlings were transferred to 1/2 MS medium supplemented with 0 or 300 mM mannitol, 175 mM NaCl, and 1.5 µM ABA and grown for different days. After 3 days, the rosette leaves were sampled for qRT-PCR and after 14 days the photographs were taken.

### 2.7. H_2_O_2_ Content, Lipid Peroxidation, and Antioxidant Enzymes

According to the method of Ding et al. [23], the lipid peroxidation of cell membrane was evaluated by the determination of malondialdehyde (MDA). The amount of MDA-TBA (thiobarbituric acid) complex was calculated from the coefficient of absorbance (155 mM^−1^ cm^−1^). The content of H_2_O_2_ was estimated by monitoring the absorbance of the titanium peroxide complex at 415 nm. Next, 0.2 g of plant material were homogenized in 1.5 mL 50 mM phosphate buffer solution (PBS) (pH 7.5) containing 0.2 mM ethylenediamine tetra acetic acid (EDTA) and 2% polyvinylpyrrolidone (PVP). The homogenate was centrifuged in a refrigerated centrifuge at 13,000× *g* for 20 min and the supernatants were used for protein determination and enzyme assay. All the steps were carried out at 4 °C. superoxide dismutase (SOD) activity was measured by monitoring the inhibition of photochemical reduction of nitroblue tetrazolium (NBT) at 560 nm. The catalase (CAT) activity was determined by measuring the decrease in absorption of H_2_O_2_ at 240 nm.

### 2.8. RNA Isolation and qRT-PCR

Total RNA was extracted with an RNA Isolater Total RNA Extraction Reagent (Vazyme Biotech Co., Ltd., Nanjing, China). Reverse transcription was performed using the HiScript II Q RT SuperMix for qPCR (Vazyme, Nanjing, China). The primer pairs used in this study are all listed in Table S1. The qRT-PCR was performed on an ABI7500 instrument (Applied Biosystems, Foster, CA, USA) using SYBR Green qPCR kits (TaKaRa, Kusatsu, Japan). The PCR amplification conditions included an initial heat-denaturing step at 95 °C for 3 min, followed by 40 cycles of 30 s at 95 °C, 30 s at 58 °C, and 1 min at 72 °C. Relative mRNA levels were normalized to that of the internal reference genes. Each sample was divided into 3 biological replicates. The data were processed on the basis of the 2^−ΔΔCT^ method.

## 3. Results

### 3.1. Genome-Wide Identification of BAG Gene Family in Tomato

The released whole-genome sequences of *Solanum lycopersicum* (ITAG2.4, 3.0, 4.0) were used in the present study. Firstly, we performed a keyword search using the BAG domain file (PF02179) as a query and 10, 14, and 12 genes were obtained from three databases, respectively. Secondly, we searched the genome protein sequence databases ITAG4.0 by BLASTP using seven Arabidopsis (AtBAG) proteins [4], and 11 gene-encoding proteins were identified. After InterProScan search and manual analysis to remove false positive and redundant genes, a total of 10 non-redundant, full-length BAG genes in *Solanum lycopersicum* were identified and designated as *SlBAG1*-*SlBAG10* based on their physical location. Interestingly, there were great differences in the information of the ten *SlBAG* genes in the three databases (Table S2). In order to further confirm the gene information, we cloned the full length of these genes to confirm the CDS sequence. Combining the sequence alignment through the NCBI database, the gene information was finalized (Table 1), which was partly different from the existing ITAG database and the reported characteristics of tomato *SlBAG* genes [24] to some extent. Compared with the BAG gene family reported by [24], there were differences in the genome sequences of eight genes and the coding protein sequences of five genes. In particular, the information of *SlBAG4* was completely different from the existing database. Therefore, we re-analyzed some characteristics of 10 *SlBAG* genes in the following sections.

These *SlBAG* genes were distributed on six chromosomes. *SlBAG1*, *SlBAG4*, and *SlBAG8* were located on chromosome 1, 4, and 10, respectively. Three *SlBAG* genes were on chromosome 6, and the rest were distributed on the other two chromosomes. The relevant characteristics of genes and proteins of SlBAGs were shown in Table 1. The range of coding sequence (CDS) was 513 bp to 3708 bp, and the range of protein was 170 to 1235 amino acids, with an average of 402 amino acids. The molecular weight range of SlBAG protein was 19.43–137.35 kDa and the isoelectric point range was 5.09–10.26.

### 3.2. Phylogenetic Characterization of BAG Family Genes in Tomato

**Figure 1:**
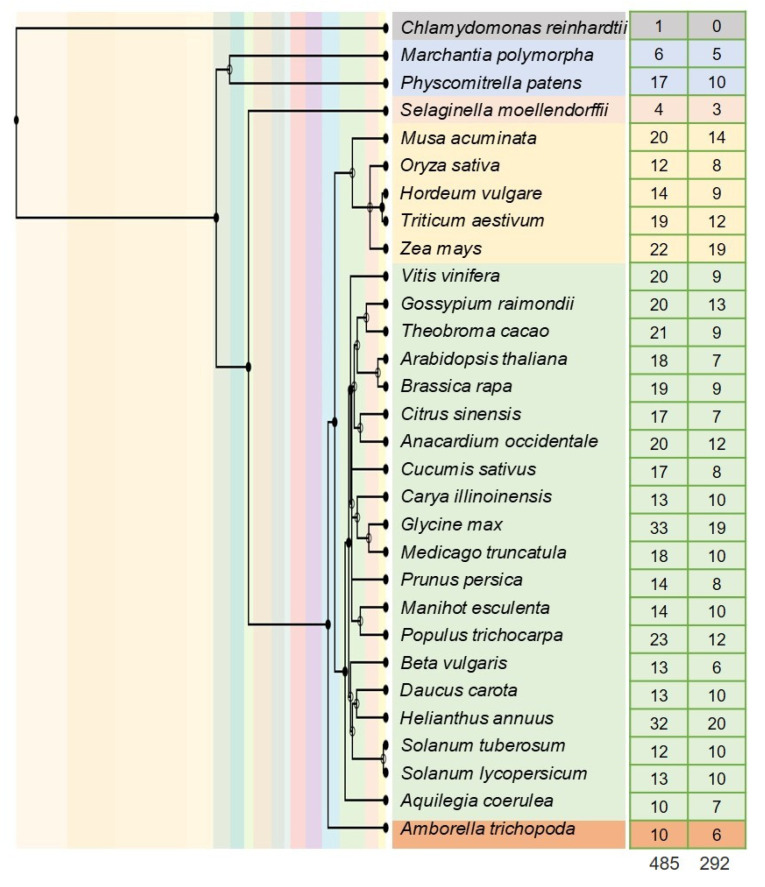
The number of BAG family in 30 plant species. The number of BAG proteins in 30 plant species and each group were shown on the right. The whole genomic data come from Phytozome 13. The number of BAG conserved domain analyzed by batch CD search were shown on the right. The species evolution tree was constructed by TimeTree 5 Beta (http://www.timetree.org, accessed on 10 January 2022).

**Figure 2:**
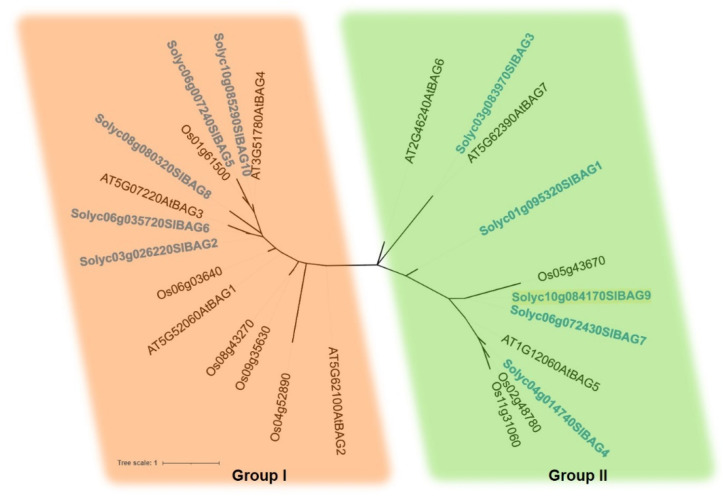
Phylogenetic tree of tomato SlBAG proteins. The 25 amino acid sequences of three plant species (Sl, *Solanum lycopersicum*; Os, *Oryza sativa*; At, *Arabidopsis thaliana*) were generated using the PhyML in MEGA 7.0. Based on the phylogenetic data, these proteins are divided into two distinct sub-groups, which are distinguished with different colors.

A comprehensive search was conducted for plant lineages, including 30 representative monocotyledons, eudicotyledons, basal angiosperms, ferns, bryophytes, and algae. As a result, 485 putative genes were identified from 30 plants with BAG as the keyword in the phytozome13 database, and 292 putative genes encoding BAG proteins were further identified by batch CD search, which were divided into five different groups (Figure 1). By now, some functions of the BAG family members have been studied in Arabidopsis and rice [7,25]. To predict the function of SlBAG proteins, the evolutionary relationship among *Arabidopsis thaliana* (7 genes), *Oryza sativa* (8 genes), and *Solanum lycopersicum* (10 genes) was constructed using the MUSCLE and PhyML method. The phylogenetic tree showed that these BAG proteins were divided into two groups (I, II) (Figure 2). Group I contained 14 members (with four, five, and five members of Arabidopsis, rice, and tomato, respectively). Cluster II included eleven members (with three, three, and five members of Arabidopsis, rice, and tomato, respectively). This classification is basically similar to the previous analysis of BAG proteins in Arabidopsis [3,21].

### 3.3. Exon–Intron Arrangement and Conserved Motifs Analysis

**Figure 3:**
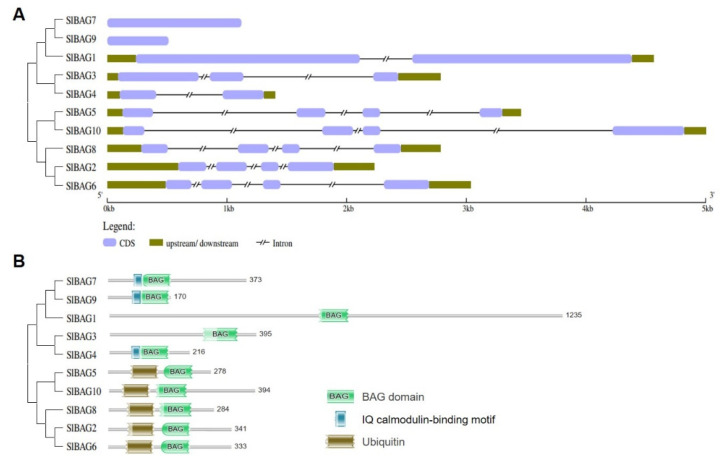
Gene structure and BAG domain of SlBAG family in tomato. (**A**) The gene structure was generated using a Gene Structure Display Server (GSDS version 2.0). The length of exons as well as introns and UTRs of each *SlBAG* gene are displayed proportionally. (**B**) BAG domains were generated through the HMMER web server (Biosequence analysis using profile hidden Markov Models|HMMER). The legends of BAG domain, Ubiquitin-like domain and IQ calmodulin-binding motif are listed on the right. The phylogenetic tree was constructed referring to Figure 2. SlBAG proteins are listed on the left.

Gene structure diagrams of *SlBAG* genes, including the presence of exon-intron components, were constructed by the Gene Structure Display Server (GSDS) (Figure 3A). As a result, new gene structural sequences were presented in this study in addition to *SlBAG7*, and *SlBAG9*, which were reported [24]. *SlBAG7* and *SlBAG9* were predicted to exhibit no introns and two *SlBAG* genes (*SlBAG1*, *SlBAG4*) contained one intron, whereas five *SlBAG* genes in group II exhibited three introns. Besides, several conserved motifs among the SlBAG proteins were calculated using the MEME, a tool used to discover motifs in a group of related DNA or protein sequences (Figure 3A). The BAG domain was analyzed using the HMMER web server (Figure 3B). Besides the BAG domain, all members of group II (SlBAG2, 5, 6, 8, 10) contained ubiquitin-like domains similar to some animal counterparts. However, SlBAG4, SlBAG7, and SlBAG9 of group I contained an IQ calmodulin-binding motif located in the N terminus, which was unique to plants. The previous study showed that the SlBAG4 has no IQ calmodulin-binding motif [24]. However, here we proved that its gene and protein structure were completely different from that in the existing database.

### 3.4. Organ-Specific Expression Profiles of SlBAG Genes

**Figure 4:**
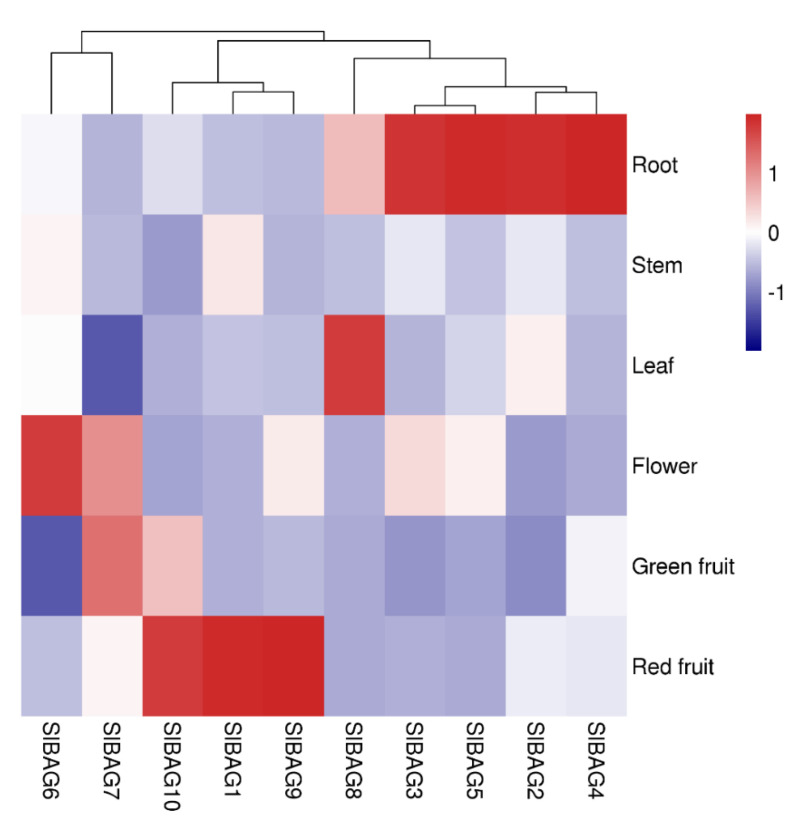
Expression profiles of *SlBAG* genes in different organs of tomato. The heatmap shows the expression change of ten *SlBAG* genes. Total RNA was extracted from different organs (root, stem, leaf, flower, green fruit, and red fruit) for qRT-PCR. Three independent biological repeats were performed (*n* = 3). The bar in the upper right corner represents the expression value of log2, and the change of expression level is represented by the change of color. Red indicates relatively high expression and blue indicates relatively low expression.

The promoters of all ten *SlBAG* genes contained different cis elements for plant growth regulation, stress responses, hormone responses, and light responses (Figure S1; Table S3). To evaluate the function of *SlBAG* genes in the growth and development, the expression pattern of ten *SlBAG* genes in different organ (root, stem, leaves, flowers, green fruits, and red fruits) was determined with qRT-PCR. The expression of *SlBAG* genes, shown as a cluster heat map, was different in various organs and some genes were only expressed in specific organs (Figure 4). *SlBAG2*, *SlBAG3*, *SlBAG4*, and *SlBAG5* were highly expressed in roots. *SlBAG8* displayed the high expression in roots and leaves. For flowers and fruits, *SlBAG6* showed the highest transcript level. *SlBAG7* showed relatively higher expression in flowers and green fruits. However, *SlBAG1*, *SlBAG9*, and *SlBAG10* showed the highest level in red fruits. These results suggest that *SlBAG* genes might be involved in the growth and development of tomato.

### 3.5. Expression Pattern of SlABG Genes under Abiotic Stress

**Figure 5:**
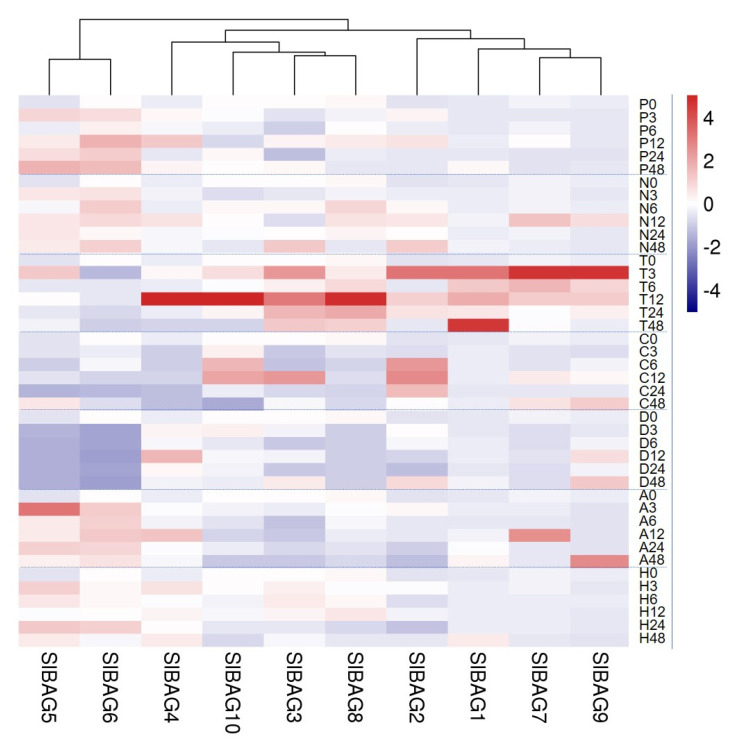
Expression profiles of *SlBAG* genes in tomato seedlings under different abiotic stresses. 20-day-old tomato plants were treated with PEG6000 (P, 20%), NaCl (N, 100 mM), high-temperature (T, 42 °C), cold (C, 4 °C), cadmium (D, 50 uM), ABA (A, 100 uM), or H_2_O_2_ (H, 10 mM) for 0, 3, 6, 12, 24, and 48 h. Total RNA from tomato leaves at indicated time was extracted from different organs (root, stem, leaf, flower, green fruit, and red fruit) for qRT-PCR. Three independent biological repeats were performed (*n* = 3). The bar in the upper right corner represents the expression value of log2, and the change of expression level is represented by the change in color. Red indicates relatively high expression and blue indicates relatively low expression.

The promoter analysis of *SlBAG* genes indicated that these genes might be related to the response of tomato to different stresses. Here, the *SlBAG* gene expression of tomato leaves after osmotic stress (drought and salt), temperature stress (high temperature and cold) and signal molecular (ABA and H_2_O_2_) was analyzed by qRT-PCR (Figure 5). Under all these stress conditions, almost all *SlBAG* genes showed different expression patterns. Under drought treatment, the expression of *SlBAG5* and *SlBAG6* generally increased with prolonged treatment time, while *SlBAG9* expression decreased with time under drought stress. Under salt stress, the expression of *SlBAG1*, *SlBAG2*, *SlBAG5*, and *SlBAG6* was upregulated. In response to high temperature, the expression level of *SlBAG1*, *SlBAG2*, *SlBAG3*, *SlBAG7*, *SlBAG8*, and *SlBAG9* in general were upregulated, while *SlBAG6* was downregulated. For cold stress, the expression levels of *SlBAG1* and *SlBAG2* in general were increased, while *SlBAG4*, *SlBAG6* and *SlBAG8* was decreased. As far as ABA is concerned, the expression of *SlBAG1*, *SlBAG5*, and *SlBAG6* generally showed an upward trend, while the expression of *SlBAG2*, *SlBAG8*, and *SlBAG10* showed a downward trend. For H_2_O_2_, the expression of *SlBAG1*, *SlBAG4*, and *SlBAG5* in general increased while expression of *SlBAG7*, *SlBAG9*, and *SlBAG10* decreased. The cluster heat map of all treatment showed that the expression of *SlBAG5* and *SlBAG6* induced by osmotic stress and signal molecules clustered in the same group and most of the *SlBAG* genes could be regulated by high temperature.

### 3.6. Heterologous Expression of SlBAG9 Conferred Sensitivity to Drought, Salt, and ABA

**Figure 6:**
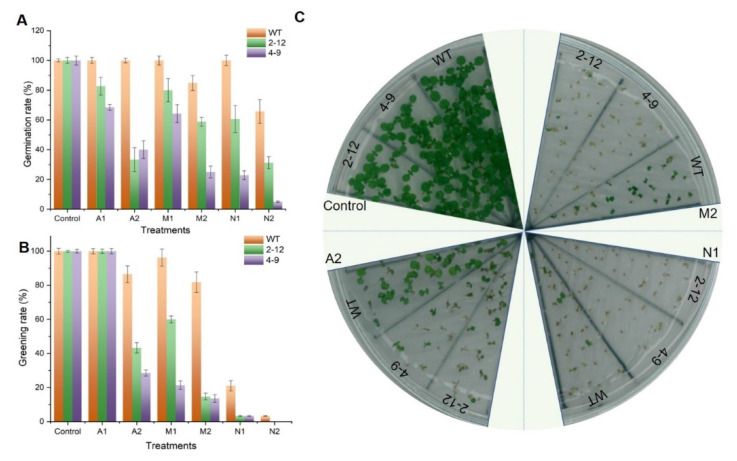
The increased sensitivity of *SlBAG9*-overexpressing Arabidopsis to mannitol, NaCl, and ABA stress. (**A**) The germination rate of *SlBAG9*-overexpressing lines (2–12 and 4–9) and wild type (WT) on 1/2 MS medium containing 200 and 300 mM mannitol (M1, M2), 175 and 200 mM NaCl (N1, N2), and 1.0 and 1.5 µM ABA (A1, A2) treatments for 5 days. (**B**) Cotyledon greening rate of *SlBAG9*-overexpressing lines and WT on 1/2 MS medium containing 200 and 300 mM mannitol (M1, M2), 175 and 200 mM NaCl (N1, N2), and 1.0 and 1.5 µM ABA (A1, A2) treatment for 10 days. Error bars indicate the SD of three replicated experiments. (**C**) The seedling growth of *SlBAG9*-overexpressing lines and WT Arabidopsis on 1/2 MS plates containing 300 mM mannitol (M2), 175 mM NaCl (N1), and 1.5 µM ABA (A2) treatments for 10 days.

Our previous studies showed that *SlBAG9* with a higher expression level at the transcriptional and protein levels in response to high temperature stress was involved in the negative regulation of thermotolerance [22,23]. The expression analysis of *SlBAG9* showed that it might be involved in the response to drought, salt, and ABA (Figure 5). To further investigate the biological functions of *SlBAG9*, heterologous overexpression lines of slbag9 in Arabidopsis (2–12, 4–9) were used here, which have been shown to be two homozygous lines [22]. We evaluated the seed germination and growth on 1/2 MS medium containing 200 and 300 mM mannitol, 175 and 200 mM NaCl, and 1.0 and 1.5 µM ABA treatments. No significant difference between the overexpression lines and WT was observed on 1/2 MS medium without treatment. However, the seed germination rate and seedling growth of *SlBAG9*-overexpressing lines were inhibited by mannitol, salt, and ABA compared to that of WT (Figure 6, Figure S2). For germination, after five days on MS medium containing 300 mM mannitol, about 85% of wild-type seeds, but less than 60% and 30% of the transgenic seeds (2–12, 4–9, respectively) germinated (Figure 6A). After ten days, more than 90% wild-type cotyledons, but less than 15% transgenic cotyledons turned green (Figure 6B), and the post-germination growth of transgenic materials was much worse than wild-type ones (Figure 6C). Two different treatments (200 and 300 mM mannitol) showed a certain concentration effect (Figure 6). For seedling growth, seven-day-old seedlings of wild-type, 2–12, and 4–9 were transferred to 1/2 MS containing 300 mM mannitol for 14 days and the growth pattern was observed. Under normal conditions, no significant difference was found in the aspects of seedlings growth of all the seedlings. Drought inhibited the growth of all seedlings, but the inhibition degree of 2–12, and 4–9 lines was greater than that of wild-type seedlings (Figure S2). A similar phenotype was also observed in NaCl treatment (Figure 6, Figure S2). These results demonstrated that *SlBAG9* conferred plant more sensitive to drought and salt stress, negatively regulating osmotic stress.

ABA is an important mediator of drought and salt and senescence [26]. To further study whether the ABA was involved in *SlBAG9*-mediated response, we observed the phenotype of *SlBAG9*-overexpressing lines grown under ABA treatment. In total, 100% of wild-type seeds, but about 40% transgenic seeds germinated after five days on 1/2 MS medium supplemented with 1.5 µM ABA (Figure 6A). More than 80% cotyledons of wild-type cotyledons, but less than 50% of transgenic lines turned green as a result of ABA treatment for seven days (Figure 6B,C). For seedling growth, ABA treatment decreased leaf chlorophyll content, while the leaves of the WT exhibited an obvious stay-green phenotype (Figure S2).

### 3.7. SlBAG9 Downregulated Stress/ABA-Responsive Genes

**Figure 7:**
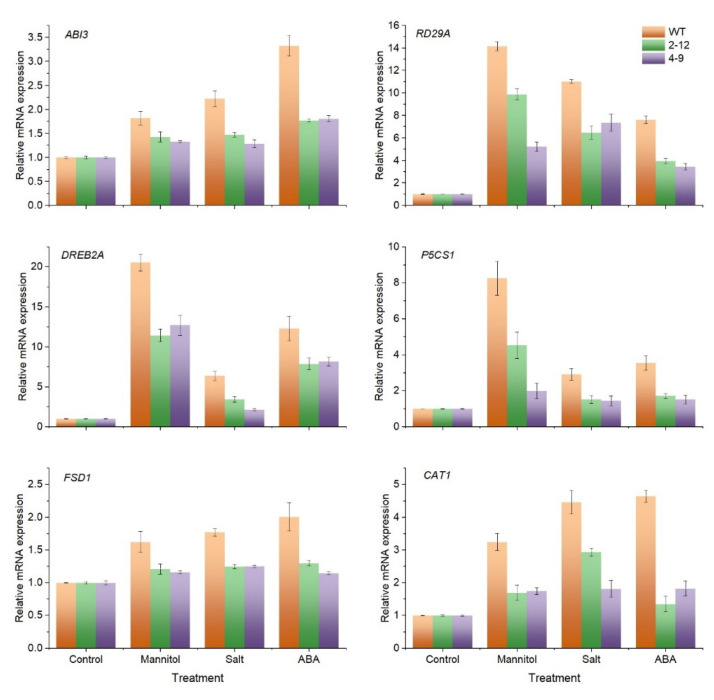
Relative expression of stress/ABA-responsive genes in *SlBAG9*-overexpressing Arabidopsis exposed to mannitol, NaCl, and ABA stress. The seven-day-old Arabidopsis plantlets of wild-type, 2–12, and 4–9 were transferred to 1/2 MS containing 0, 300 mM mannitol, 175 mM NaCl, and 1.5 µM ABA for three days. Total RNAs were extracted, and qRT-PCR analyses were performed. Error bars indicate the SD of three replicated experiments.

To better understand the mechanism of *SlBAG9*-mediated osmotic stress, mRNA levels of two ABA-responsive genes *ABI3*, and *RD29A* and four stress-responsive genes *DREB2A*, *P5CS1*, *FSD1*, and *CAT1* were determined by qRT-PCR. The RNA transcript levels of the six genes were not significantly altered under normal growth conditions (Figure 7). However, the expression levels of these genes were upregulated in both WT and transgenic plants under osmotic stress or ABA treatment, but the increase was not as pronounced in transgenic plants as in WT plants (Figure 7). These results suggested that these genes in the *SlBAG9*-overexpressing plants are more sensitive to osmotic stress than WT plants, and that *SlBAG9* may be involved in the regulation of stress-regulated gene expression. The expression levels of two key ROS scavenging enzyme gene *FSD1*, and *CAT1* were also regulated by *SlBAG9*, indicating that *SlBAG9* modulates the ROS scavenging system. In view of this, it was speculated that the ROS scavenger-related enzyme activity was regulated by *SlBAG9*. Therefore, in the next section we further analyze the possible effect of *SlBAG9* on the oxidative damage and the activities of SOD and CAT.

### 3.8. SlBAG9 Overexpression Aggravated Oxidative Damage under Drought, Salt, and ABA

**Figure 8:**
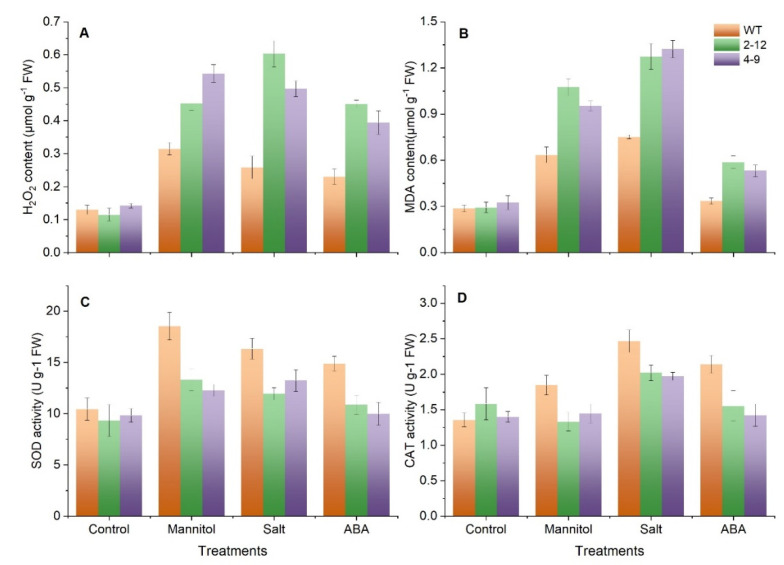
*SlBAG9* overexpression aggravated oxidative damage in Arabidopsis under mannitol, NaCl, and ABA treatment. The seven-day-old Arabidopsis plantlets of wild-type, 2–12, and 4–9 were transferred to 1/2 MS containing 0, 300 mM mannitol, 175 mM NaCl, and 1.5 µM ABA for three days for physiological change evaluation. The H_2_O_2_ content (**A**), MDA content (**B**), SOD activity (**C**), and CAT activity (**D**) were analyzed quantitatively in WT and SlBAG9-overexpressing lines 2–12 and 4–9. Error bars indicate the SD of three independent experiments.

Stress conditions trigger the accumulation of ROS molecules, resulting in oxidative damage to plant organelles [27]. To uncover whether *SlBAG9* was involved in drought, salt, and ABA-induced oxidative stress, the H_2_O_2_ level was estimated. Excessive accumulation of H_2_O_2_ was observed in the *SlBAG9*-overexpressing lines compared with the wild-type plants under drought, salt, and ABA conditions (Figure 8A). MDA is considered to be an effective marker of membrane damage caused by oxidative stress [28]. In accordance with the stressed phenotype, *SlBAG9*-overexpressing line 2–12 and 4–9 accumulated more MDA compared with the wild type under drought, salt, and ABA treatment (Figure 8B). These results indicated that *SlBAG9* can sensitively respond to drought, salt, and ABA to induce H_2_O_2_ generation and cause membrane lipid peroxidation, resulting in hypersensitivity responses. Since the above data suggested that *SlBAG9* mediates oxidative damage, and downregulate ROS scavenger-related gene *FSD1* and *CAT1*, the activities of SOD and POD were determined. There was little difference in SOD and CAT activities in WT, 2–12, and 4–9 genotypes under normal conditions. However, the SOD and CAT activities were lower in the 2–12 and 4–9 lines compared with the WT (Figure 8C,D). This result indicated that *SlBAG9* overexpression is implicated in ROS clearance and reduces tolerance to drought, salt, and ABA-induced oxidative stress by regulating antioxidant enzyme activity, which could support different change patterns of H_2_O_2_ and MDA (Figure 8).

## 4. Discussion

Cultivated tomato is easily affected by various environmental factors. Researchers have been studying potential resistance genes in plants for a long time. With the available tomato genome, the study of gene function is becoming more and more important [8]. Our previous research made us very interested in the tomato *SlBAG* gene family [22,29]. Many studies have shown that *BAG* gene plays an important role in plant growth, development, and stress response (reviewed by Thanthrige et al. [7]). Therefore, it is necessary to identify *SlBAG* genes and their biological function in tomato. Lately, Irfan et al. [24] identified 11 BAG genes using the tomato database (ITAG2.4). However, the database now includes three versions (2.4, 3.0, 4.0), in which different BAG gene information is presented. The present study aimed to a comprehensive genome-wide functional characterization of *SlBAG* genes and proteins in tomato. We obtained 10 BAG genes through sequence alignment and multi database comparison, cloned all ten CDS sequences, and finally determined the information of these genes (Table 1 and Table S2). Compared with the newly published genetic information obtained by Irfan et al. [24], *SlBAG6*, *SlBAG8*, *SlBAG10*, *SlBAG4*, and *SlBAG1* have new CDS sequences and eight gene structural sequences were new in addition to *SlBAG7*, and *SlBAG9*.

For the ten BAG proteins in this study, their protein structures and evolutionary relationships with BAG proteins in tomato, Arabidopsis and rice were analyzed. The proteins were divided into two groups, and the difference is mainly at their N-terminal. In addition to BAG domain, the first group has a UBL domain. A UBL domain can interact with 26S proteasome and is an indispensable part of BAG1 in stress response [30]. UBL domain in the group I suggests that they may also participate in the degradation of some proteins as molecular bridges. The second group has a specific CaM-binding domain IQ motif near the BD domain in plants, indicating that it may be involved in unique biological processes [31]. Irfan et al. [24] found that SlBAG7 and SlBAG9 had IQ domain. In the present study, three SlBAGs (SlBAG4, SlBAG7, SlBAG9) in the group II have IQ domain, indicating that their biological functions may be related to the Ca^2+^ signal. IQ motif can bind CaM and affect the formation of complex between CaM and targeted protein [32]. In vitro studies have shown that Ca^2+^ can affect the binding affinity of AtBAG6 and CaM and regulate the process of cell death mediated by AtBAG6. The CaM-binding motif and the BD are required for AtBAG6-mediated cell death [32]. As a signaling hub, AtBAG5 connects the Ca^2+^ signaling network with the Hsc70 chaperone system to regulate plant senescence. The IQ motif mutant retains the association between AtBAG5 with Hsc70 while disrupting the association of AtBAG5 with apo-CaM [11]. It is possible that the increase in Ca^2+^ in the mitochondrial matrix may protect mitochondria from senescence through a combination of Ca^2+^ and apo-CAM, so as to promote the release of Hsc70 from CaM/AtBAG5/Hsc70 signal complex and to inhibit ROS production [11,12].

The BAG family is widely distributed in the plant kingdom (Figure 1). The BAG family also exists in various plant tissues and organs. Results from many experiments have shown that the BAG gene family plays an important role in plant growth and development [3]. *AtBAG4* and *AtBAG6* expressions were detected in the root, stem, leaf, and flower of Arabidopsis. In the whole development process, *AtBAG2* and *AtBAG6* genes are expressed in various tissues in overlapping or specific expression pattern [33]. Arabidopsis knockout mutant *atbag4* or *atbag6* has the phenotype of early flowers and multi branching inflorescences, with a shortened life cycle and early aging. It was found that the rosette diameter of the 4-week-old *atbag2* mutant was larger than that of the wild type [34]. Arabidopsis plants overexpressing *AtBAG6* are shorter than wild-type plants [3]. The tissue-specific expression experiment of rice showed that *OsBAG1*, *OsBAG3*, and *OsBAG4* had the highest expression in roots, stems, and internodes, indicating that they may be involved in cell elongation and expansion. Rice OsBAG4 and EBR1 form a protein complex, which makes EBR1 control its protein stability level through ubiquitination of OsBAG4, inhibit growth and development, resulting in plant dwarfism [17]. Irfan et al. [24] showed that several *SlBAG* genes such as *SlBAG1*, *SlBAG3*, *SlBAG6*, and *SlBAG9* had differential expression during fruit development, which suggested that they might have a role in fruit development as well. In this study, many *SlBAGs* in tomato showed specific expression patterns in organs, indicating their important roles (Figure 4). Recently, He et al. [8] showed that *BAG2* and *BAG5b* were highly expressed in tomato stem and flower. Here, the corresponding name was *SlBAG7* and *SlBAG9*, respectively, which was not only highly expressed in flowers, but also in fruits (Figure 4).

The regulatory elements in plant promoters play an important regulatory role at the transcriptional level [35]. Here, it was found that in all *SlBAG* promoters there are a series of elements related to abiotic stress and hormones, such as MYC, MBS, DRE, HSE, W-box, and ABA and SA- responsive cis-elements (Figure S1). These results were consistent with the previous study that most of the same cis elements were found in 2000 bp upstream regions of *SlBAG* genes [24] and ~1000 bp upstream regions of all Arabidopsis BAG family genes, suggesting that they play a role in coping with different environmental stresses such as cold, drought and high salinity [4]. Take W-box as an example, AtBAG7 translocates from the ER to the nucleus, where it interacts with the transcription factor WRKY29, which then binds to the W-box in the promoter of *AtB*AG7 to initiate the transcription of *AtBAG7* and other chaperones to promote stress tolerance [36]. The accumulated evidence shows that BAG expression can be regulated by various abiotic stresses [3,7,24,36]. Accordingly, here we monitored the transcriptional response of tomato *SlBAG* genes with the exposure to various stress conditions including drought, salt, HT, cold, as well as ABA and H_2_O_2_ signals (Figure 5). Plant *BAG* gene expression is related to its function to some extent. Cold upregulated the expression of *AtBAG4* and *AtBAG4* overexpression increased tobacco plants tolerance to cold, salt, UV, and oxidative stress [3]. Heat stress significantly upregulated *AtBAG6* gene and protein level and the *atbag6* mutant is sensitive heat stress [4,33]. Transgenic rice plants overexpressing salt-induced *OsBAG4* showed tolerance to NaCl stress [25]. *AtBAG6* transcript levels were significantly upregulated by H_2_O_2_ [3]. The expression of several *SlBAG* genes was also induced by ACC, the precursor of ripening hormone ethylene and ABA, suggesting that *SlBAG* genes are potentially involved in the fruit ripening regulation and stress response [24]. In this study, some *SlBAG* genes also showed similar expression patterns. However, the involvement of H_2_O_2_ in the BAG-mediated biological function is unclear. The above results suggested that *SlBAG* family is involved in the response of tomato plants to abiotic stresses such as salt, drought, cold and HT, and ABA and H_2_O_2_ signals may be involved in these pathways.

*SlBAG9* was noteworthy because in our previous studies it showed higher gene expression level and protein abundance under high temperature stress [22]. Overexpression of *SlBAG9* decreased the tolerance to HT [22,29]. Cis-elements and expression analysis indicated that *SlBAG9* may be involved in drought, salt, and ABA stress (Figure S1 and Figure 5). This study further showed that *SlBAG9*-overexpressing Arabidopsis exhibited increased sensitivity to mannitol, salt, and ABA treatment (Figure 8 and Figure S1). In terms of salt stress, many studies have shown that BAG can positively regulate plant salt tolerance. Arabidopsis with overexpression of *TaBAG* and *TaBAG2* showed significant enhancement of salt tolerance [6]. The *atbag4* mutant was more sensitive to salt stress [3]. Transgenic rice plants heterologously expressing *AtBAG4* showed higher salt tolerance than WT [37]. Recently, Wang et al. [25] reported that OsBAG4 functioned as a bridge between OsMYB106 and OsSUVH7 under salt stress to regulate OsHKT1;5 expression, so as to improve salt tolerance. Pan et al. [38] showed that salt suppressed *BAG6* and *BAG7* expression but addition of ACC (1-aminocyclo-propane-1-carboxylic acid) in the salt treatment could re-activate *BAG6* and *BAG7* expression, indicating that BAG genes are involved in the process of plant cell death induced by salt stress. The negative regulation mechanism of *SlBAG9* on salt tolerance remains to be studied. As far as drought and ABA are concerned, there are few studies on BAG gene function so far. Arabidopsis leaves with low-level *AtBAG4* overexpression appeared to be drought tolerant [3]. AtBAG4 interacted with potassium influx channel protein KAT1 in guard cells to regulate stomatal movement [39]. However, our observation was consistent with the latest data from Arabidopsis *AtBAG2* and *AtBAG6* [33]. Germination of *atbag2*, *atbag6*, and *atbag2atbag6* seeds was less sensitive to ABA compared to WT, whereas *AtBAG2* and *AtBAG6* overexpression lines showed the opposite results for ABA. The survival rate of *atbag2*, *atbag6*, and *atbag2atbag6* plants was higher than that of the WT under drought stress. In addition, these mutants showed differential expression of several stress and ABA-related genes and low ROS levels after drought and ABA treatment [33]. In this study, *ABI3*, *RD29A*, *DREB2A*, and *P5CS1* expression of transgenic plants was lower than that of WT plants under osmotic stress. In *Arabidopsis thaliana,* these genes had all been shown to be inducible by drought, salinity, or ABA. The higher expression of these stress genes was related to plant tolerance [40]. All these data indicated that the decreased transcription levels of these genes in *SlBAG9*-overexpressing lines lead to enhanced sensitivity to osmotic stress, which might be mediated by ABA. There are three points of view that support our hypothesis. First, the *SlBAG9* expression was induced by exogenous ABA (Figure 6). Second, *SlBAG9* overexpression in Arabidopsis resulted in hypersensitivity to ABA (Figure 7 and Figure S2). Third, under ABA and osmotic stress, the *SlBAG9* overexpression significantly downregulated the expression levels of ABA signaling pathway genes such as *ABI3* and *RD29A* (Figure 8), which were reported to play positive regulators in ABA-associated abiotic stress [41]. The decrease in *ABI3* and *RD29A* expression of *SlBAG9* overexpression lines might be one of the reasons for the increased sensitivity to ABA and osmotic stress. The specific signaling pathway of ABA mediated by *SlBAG9* remains to be unveiled.

It is known that a variety of environmental stresses can promote ROS production and the senescence process will also increase the accumulation of ROS. Therefore, the regulation of ROS production plays a key role in senescence and stress response [42]. It has been reported that *atbag2*, *atbag6*, and *atbag2atbag6* seedlings lines accumulated lower H_2_O_2_ when treated with drought and ABA [33]. The transgenic plants overexpressing *AtBAG5* showed greater H_2_O_2_ accumulation than WT, indicating that *AtBAG5* is involved in leaf senescence by regulating the production of ROS. In the present study, overexpression of *SlBAG9* in Arabidopsis could induce the excess production of H_2_O_2_ in response to drought, salt, and ABA (Figure 8), suggesting that *AtBAG5* may be involved in regulating these stress responses though the proliferation of ROS. Excessive production of ROS will lead to oxidative stress and cell death in growing plants [43] H_2_O_2_ and MDA content is recorded with the extent of oxidative stress [28]. Consistent with the results of H_2_O_2_, *SlBAG9*-overexpressing seedlings exhibited significantly induced H_2_O_2_ and MDA accumulation compared to the WT seedlings after stress treatment. (Figure 8). These results corresponded well with the phenotype of these *SlBAG9* overexpression plants in response to drought, salt, and ABA, which suggested that *SlBAG9* accelerate H_2_O_2_ excess production, leading to a deeper degree of oxidative damage. Correspondingly, gene expression analyses showed *SlBAG9* overexpression downregulated the expression of key ROS scavenger-related genes *FSD1* and *CAT1* (Figure 7). *FSD1* encodes a chloroplast/nuclei/cytosol localized SOD that utilizes Fe as the cofactor (FeSOD) and FSD1 presents osmoprotection in Arabidopsis [44]. *CAT1* encodes a peroxisomal catalase (CAT1), which is implicated in the drought and salt stress responses [45]. SOD and CAT scavenge ROS by converting superoxide to H_2_O_2_ and H_2_O_2_ to oxygen and water, sequentially. There is a closely correlation between the expression of both two genes and enzyme activity. Li et al. [46] found that the expression of *CAT1* and *FSD1* were upregulated in *PpDHN*-overexpressing plants under drought and salt stress and elevated levels of SOD and CAT enzyme activities were detected accompanying the trends in expression of these genes. In the transgenic overexpressing *IpASR*, *CAT1* and *FSD1* expression and SOD and CAT enzyme activities showed the same trend under salt and drought stress [47]. In the current study, it was clearly shown that the expression of *FSD1* and *CAT1* (Figure 7) and the activities of SOD and CAT (Figure 8C,D) were not altered among wild-type and over-expressing *SIBAG9* lines; however, a difference was only detected under stress conditions or in response to exogenous ABA supply. Taken together all the above indicate that *SIBAG9* is involved in regulation of stress responses, which needs to be further explored. ABA signaling pathway is an important way for plants to deal with abiotic stress and ABA also regulates senescence [26]. Whether ABA was involved in SlBAG9-mediated antioxidant protection pathway still needed further study. Taken together, it was suggested that the increased sensitivity of *SlBAG9* overexpression to drought, salt, and ABA might be related to the oxidative damage regulated by antioxidant enzyme system such as SOD and CAT. This complex mechanism needs to be deeply investigated in the future.

## 5. Conclusions

In the present study, we identified and characterized ten *SlBAGs* at tomato genome level, which were supported by structural characteristics of the SlBAG proteins and genes, as well as by phylogenetic analysis. Large-scale expression of these *SlBAGs* by qRT-PCR further revealed tissue- and abiotic stress-specific expression patterns, which might be related to plant growth and abiotic stresses. Additionally, *SlBAG9* conferred sensitivity to osmotic stress when heterologously expressed in transgenic Arabidopsis. The enhanced sensitivity was ascribed to the decreased expression of several stress and ABA-responsive genes, the increased oxidative damage as well as the decreased ROS scavenging capability in the *SlBAG9*-overexpressing plants. Besides, it is worth noting that this study not only provide a foundation for understanding the functions of *SlBAG* gene family but also has important guiding significance for its final application in molecular resistance breeding.

## Figures and Tables

**Table 1:** The sequence characteristics of SlBAG family members in tomato.

Name ^a^	Gene Identifier ^b^	Gene Symbol ^c^	Gene ^d^			Protein ^e^		
			Chr	Intron	CDS (bp)	AA	MW (kDa)	pI
SlBAG1	Solyc01g095320	LOC101246665	1	2	3708	1235	137.35	5.30
SlBAG2	Solyc03g026220	LOC101264896	3	3	1026	341	38.25	9.52
SlBAG3	Solyc03g083970	LOC101246514	3	2	1188	395	45.28	9.43
SlBAG4	Solyc04g014740	LOC109119998	4	0	651	216	24.44	5.83
SlBAG5	Solyc06g007240	LOC101243790	6	3	837	278	31.53	6.28
SlBAG6	Solyc06g035720	LOC101267811	6	3	1002	333	37.56	9.45
SlBAG7	Solyc06g072430	LOC104648101	6	0	1122	373	42.50	5.66
SlBAG8	Solyc08g080320	LOC101246459	6	3	855	284	32.33	9.73
SlBAG9	Solyc10g084170	LOC101250069	10	0	513	170	19.43	10.26
SlBAG10	Solyc10g085290	LOC101258018	10	4	1185	394	41.91	5.09

^a^: Tomato BAG protein names given in this study; ^b^: gene ID in the ITAG4.0 database; ^c^: gene symbol in NCBI; ^d^: gene characteristics; ^e^: protein characteristics; CDS, coding DNA sequence; AA, amino acid; MW, molecular weight; pI, isoelectric point; kDa, kilodalton.

## Data Availability

The data presented in this study are available in the article and its Supplementary Materials.

## Supplementary Materials

The following supporting information can be downloaded at: https://www.mdpi.com/article/10.3390/antiox11030598/s1, Figure S1: Cis-elements components in the promoters of tomato *SlBAG* genes; Figure S2: The increased sensitivity of *SlBAG9*-overexpressing Arabidopsis exposed to mannitol, NaCl, and ABA stress; Table S1: Primers used in this study for qRT-PCR analysis; Table S2: Information of 10 *SlBAG* genes from multiple data sources; Table S3: The cis-element of *SlBAG* gene promoters.
