# Supplementary material for: Genome-Wide Identification of the Bcl-2 Associated Athanogene (BAG) Gene Family in Solanum lycopersicum and the Functional Role of SlBAG9 in Response to Osmotic Stress
Source: Antioxidants (Basel). 2022 Mar 21;11(3):598. doi: 10.3390/antiox11030598 (PMC8945447; doi:10.3390/antiox11030598)
Supplement: Supplementary file 1 [file antioxidants-11-00598-s001.zip › antioxidants-1620137-SM figure S1.pdf]

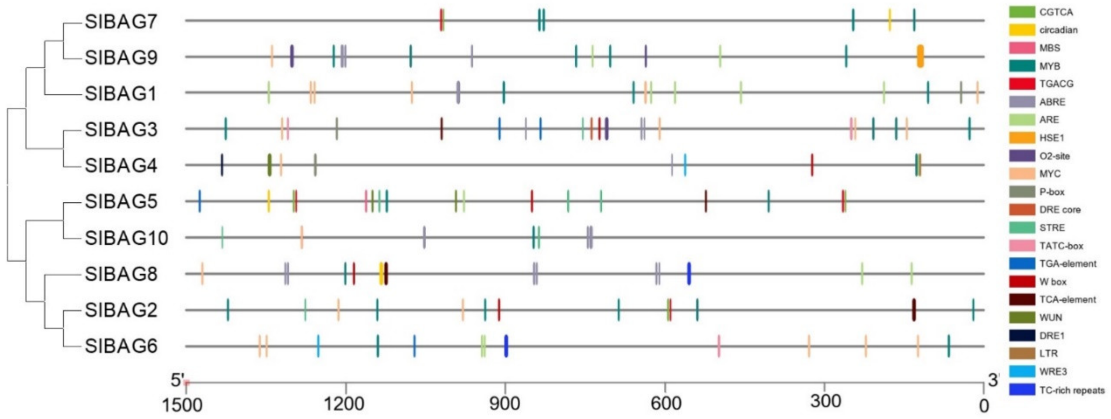

Figure S1. Cis-elements components in the promoters of tomato *SlBAG* genes. The putative cis-regulatory elements of 1500 bp upstream of *SlBAG* genes were predicted using PlantCARE program (http://bioinformatics. psb.ugent.be/webtools/plantcare/html/), which were visualized by TBtools. All putative cis-regulatory elements of *SlBAG* genes were listed in Additional Table S3.

Promoter cis-elements analysis

In order to analyze the cis-elements that might be involved in regulating the expression of *SlBAG* genes, the 1.5 kb of 5′ flanking region upstream of each *SlBAG* gene was selected and analyzed using PlantCARE tool. The promoters of all ten *SlBAG* genes contained different cis-elements for plant growth regulation, stress responses, hormone responses, and light responses (Figure S1; Table S3). For example, MYB, a stress-responsive cis-element, was the most abundant element, which was detected in all *SlBAG* genes. DRE (Drought responsiveness) only existed in the promoters of *SlBAG3* and *SlBAG4*. HSE (heat shock element) and LTR (low-temperature responsiveness) only existed in the promoters of *SlBAG9* and *SlBAG4*, respectively. Hormone response elements specific to jasmonic acid (JA), abscisic acid (ABA), salicylic acid (SA) and gibberellin acid (GA) were found upstream of several *SlBAG* genes (Figure S1). ABA-responsive elements (ABREs) were the most abundant elements, while *SlBAG2*, *SlBAG5*, *SlBAG6*, and *SlBAG7* had no this element. Eight MeJA-responsive elements (CGTCA-motif and TGACG-motif) were detected in *SlBAG2*, *SlBAG5*, and *SlBAG7*. Six GA-responsive elements (P-box and TATC-box) were found in *SlBAG1*, *SlBAG3*, *SlBAG4*, and *SlBAG6*. Four SA responsiveness (TCA-element) were found in *SlBAG2*, *SlBAG3*, *SlBAG5*, and *SlBAG8*. The data here suggested that *SlBAG* genes might function in the plant growth, stress, and hormone responses.
