# Supplementary material for: Genome-Wide Identification of the Bcl-2 Associated Athanogene (BAG) Gene Family in Solanum lycopersicum and the Functional Role of SlBAG9 in Response to Osmotic Stress
Source: Antioxidants (Basel). 2022 Mar 21;11(3):598. doi: 10.3390/antiox11030598 (PMC8945447; doi:10.3390/antiox11030598)
Supplement: Supplementary file 1 [file antioxidants-11-00598-s001.zip › antioxidants-1620137-SM figure S2.pdf]

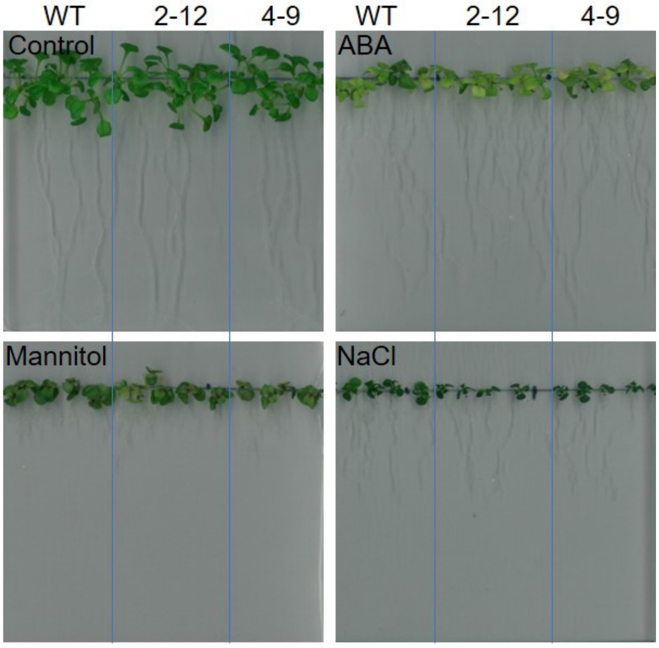

Figure S2. The increased sensitivity of *SlBAG9*-overexpressing Arabidopsis exposed to mannitol, NaCl, and ABA stress. Seven-day-old seedlings grown on the 1/2 MS media were transferred to 1/2 MS media supplemented with 0, 300 mM mannitol, 175 mM NaCl, or 1.5 μM ABA and vertically grew for 14 days.
